# Effects of Caffeine on THP-1 Myelogenous Cell Inflammatory Gene Expression

**DOI:** 10.3390/cimb47040248

**Published:** 2025-04-02

**Authors:** Zeyar T. Htun, Thomas M. Raffay, Richard J. Martin, Peter M. MacFarlane, Tracey L. Bonfield

**Affiliations:** 1 Division of Neonatology, Department of Pediatrics, New York University Grossman Long Island School of Medicine, Mineola, NY 11501, USA; 2 Division of Neonatology, Department of Pediatrics, Rainbow Babies & Children’s Hospital, Case Western Reserve University, Cleveland, OH 44106, USA; tmr12@case.edu (T.M.R.); richard.martin@uhhospitals.org (R.J.M.); pmm71@case.edu (P.M.M.); 3 Department of Genetics and Genome Sciences, Case Western Reserve University, Cleveland, OH 44106, USA; tlb7@case.edu

**Keywords:** caffeine, monocytes, inflammation

## Abstract

Caffeine is administered to preterm infants in neonatal intensive care units for prevention and treatment of apnea of prematurity. Although caffeine’s primary effect is to impact the respiratory drive of preterm infants, caffeine also has anti-inflammatory properties. This study investigated the role of caffeine on the inflammatory gene expression in THP-1 pre-monocytes exposed to lipopolysaccharide (LPS) in vitro, mimicking a clinical pro-inflammatory scenario. The effects of different physiologic dosages of caffeine administration post-LPS (treatment with caffeine) and pre-LPS (prophylaxis with caffeine) on pro-inflammatory gene expressions (*TNF-α*, *NF-κB*, *IL-8*, *PPARγ*) of the THP-1 cells were investigated. The post-LPS group showed a dose-dependent decrease in *TNF-α* at a caffeine concentration of 100 μM and *NF-κB* gene expression at 50 and 100 μM, with the implication that this is an optimal anti-inflammatory caffeine concentration range. Clinically, this would correspond to a serum caffeine level between 10 and 20 μg/mL, respectively. For the pre-LPS group, *TNF-α* and *NF-κB* gene expression decreased at all studied caffeine concentrations. These findings point to caffeine’s potential therapeutic capacity in regulating monocyte inflammatory responses to gram-negative infections in addition to regulating neuron response in the brainstem for preterm infants.

## 1. Introduction

Caffeine is used for preterm infants in neonatal intensive care units (NICUs) primarily for the prevention and treatment of apnea of prematurity. Central inhibition of adenosine receptors is one of the mechanisms of action of caffeine to prevent apnea and stimulate breathing [1]. The central inhibition stimulates the respiratory drive of preterm infants, leading to fewer apneic events and decreased exposure to mechanical ventilation, supplemental oxygen, and barotrauma [2]. Caffeine has been shown to have long-term benefits such as decreased incidence of bronchopulmonary dysplasia (BPD) and increased rate of survival without neurodevelopmental disability [2,3,4] for preterm infants. However, caffeine can also have an anti-inflammatory effect that may be beneficial to the preterm neonate, with the capacity to mitigate the immature neonatal immunity-altered responses to infection exposure [5,6].

Exposure to inflammatory environments, such as chorioamnionitis, in the preterm population, is common and can increase the risk of long-term comorbidities such as BPD. In animal models using the *Escherichia coli* endotoxin lipopolysaccharide (LPS) to mimic inflammation from chorioamnionitis and pulmonary inflammation [7,8,9], the pro-inflammatory response by macrophages inhibits normal airway morphogenesis [10]. In newborn animal models, caffeine was shown to have anti-inflammatory effects by limiting chemokine/cytokine expression [9] and was also shown to reduce the functional, architectural, and inflammatory pulmonary changes induced by inflammation secondary to hyperoxia [11].

The anti-inflammatory benefit of caffeine can impact adenosine, NF-κB, and MAPK phosphorylation, STAT1 signaling, and NLRP3 inflammasome activity [12,13,14,15]. However, immune receptiveness is likely going to be defined by caffeine dose, implicating that different caffeine concentrations likely elicit different responses, which may impact the anti-inflammatory versus adenosine receptor-driven stimulation of respiratory drive. Chavez Valdez et al. reported that caffeine treatment in preterm infants and caffeine exposure in cord blood monocytes led to changes in cytokine profiles; higher caffeine levels were associated with increased pro-inflammatory profiles, while there appeared to be an optimal caffeine level that was associated with attenuation of the pro-inflammatory profile [16,17,18]. We sought to optimize caffeine dosing as an anti-inflammatory for translation to neonate preterm care. Utilizing a homogenous pre-monocytic THP-1 human cell line, we interrogated caffeine dosing paradigms on LPS-induced pro-inflammatory gene expression in vitro to characterize the initial response to LPS in undifferentiated immune cells independent of caffeine’s capacity to stimulate respiratory drive. In our study, we analyzed the effect of pre- and post-caffeine treatments on LPS-induced THP-1 gene expression using caffeine concentrations related to clinical dosing and practice.

## 2. Materials and Methods

### 2.1. Cell Culture of THP-1 Human Pre-Monocytes

THP-1 cells (RRID:CVCL 0006) are an immortalized human pre-monocyte leukemia line obtained from the American Type Culture Collection (#TIB-202, ATCC, Manassas, VA, USA) [19]. The cells were cultured at a density of 1.0 × 10^6^ cells/uL in RPMI-1640 growth medium with 10% fetal bovine serum and 1% penicillin–streptomycin–glutamine solution and maintained at 37 °C in a 5% CO_2_ humidified incubator for maintenance. THP-1 cells were seeded into 6-well plates at 5 × 10^5^ cells/well and adhered and quiesced overnight prior to LPS (Sigma Aldrich, St. Louis, MO, USA) and caffeine (Sigma Aldrich, St. Louis, MO, USA) exposure.

### 2.2. Caffeine/LPS Exposure and Experimental Groups

The experimental groups were as follows: (1) control (only growth media, RPMI-1640), (2) LPS exposure (1 μg/mL) [20], (3) caffeine control at respective studied concentrations, and either (4a) post-LPS group (caffeine given as “treatment” after LPS exposure for thirty minutes) or (4b) pre-LPS group (caffeine given as “prophylaxis” for thirty minutes before LPS exposure). In preterm infants, a loading dose of 20 mg/kg of caffeine citrate (corresponds to 10 mg/kg of caffeine) followed by a maintenance dose of 5–10 mg/kg has been shown to achieve a serum caffeine level between 5 and 25 μg/mL [21,22]. These data were utilized to define caffeine concentrations of 25 μM, 50 μM, 100 μM, and 150 μM to directly correlate with clinical serum concentrations of 5, 10, 20, and 30 μg/mL, respectively [16]. All the experiment groups were harvested after 2.5 h to monitor the acute response of LPS and the impact of the respective caffeine concentration. The time point of 2.5 h was based on previous studies showing that LPS exposure elicited the highest TNF-α expression after two to four hours [23,24]. Cell pellets were isolated and used for RNA isolation, cDNA synthesis, and RT-PCR.

### 2.3. Gene Expression Measurements

RNA isolation, cDNA synthesis, and RT-PCR: The cell pellets were lysed with Trizol reagent and washed with chloroform. The lysed cells were centrifuged at 12,000× *g* for 15 min at 5 °C to achieve phase separation, and the RNA-containing aqueous layer was removed. Total RNA was precipitated using isopropanol, centrifuged at 12,000× *g* for 10 min at 5 °C, washed three times with 70% ethanol, centrifuged at 7500× *g* for 5 min at 5 °C, and dissolved in sterile nuclease-free water at 55 °C. RNA was reverse-transcribed into cDNA using a qScript cDNA synthesis kit (Quantabio, Beverly, MA, USA, Catalog #95048-025; Software version 1.2x by QuantStudioTM, Applied Biosystems, ThermoFisher, Waltham, MA, USA), verified for purity and concentration by spectrophotometry and stored at −20 °C. cDNA was combined with TaqMan^®^ Universal PCR Master Mix and TaqMan^®^ Gene Expression Assays (ThermoFisher, Pleasanton, CA, USA) for *PPIA* (housekeeping gene), *TNF-α*, *NF-κB*, *CXCL8* (*IL-8*), and *PPARγ*. Reactions were run on an Applied BioSystems 7300 Real-Time PCR System (Applied Biosystems software version 1.4, Foster City, CA, USA).

### 2.4. Immunoassay Measurement

Supernatants from the THP-1 were harvested when the cell pellets were harvested, centrifuged, aliquoted, and stored at −80 °C before analyzing on Luminex Multiplex (Millipore, HCYTOMAG-60K; Software: xPONENT Version 3.1, Luminex Corporation, Austin, TX, USA). The cytokine-chemokine five-plex was run using manufacturing protocols in collaboration with the CTSC Bioanalyte Core. Immunoassay for TNF-α, IL-6, IL-10, IL-1β, and IL-17 performed. Luminex (xPONENT) software analyzes the data in multiplexes using a multiplex standard curve and quality controls, with each sample being analyzed in duplicate for 50 bead events for each analyte. 

### 2.5. Statistics

Statistical analysis was performed using one-way ANOVA with post hoc Tukey test on ∆Ct values. Statistical analysis was performed using the Graph Pad Prism 10.0 software; *p* < 0.05 was considered statistical significance.

## 3. Results

### 3.1. Caffeine Effects on Constitutive Gene Expression of TNF-α, NF-κB, IL-8, and PPARγ

**Figure 1:**
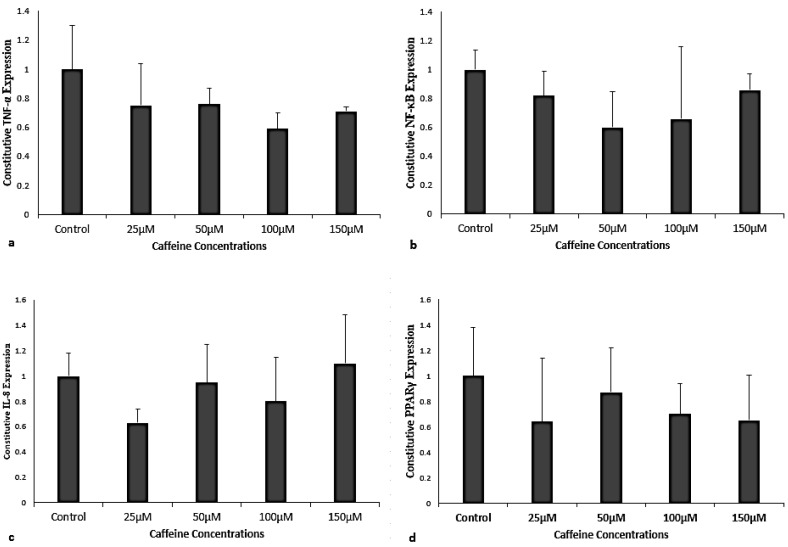
(**a**–**d**) Exclusive caffeine exposure for 2.5 h on constitutive *TNF-α*, *NF-κB*, *IL-8*, and *PPARγ* gene expression represented by fold changes (*Y*-axis). The *X*-axis shows different caffeine concentrations at 25, 50, 100, 150 μM, respectively, compared to the control, which has no exposure to caffeine. Fold change in *TNF-α*, *NF-κB*, *IL-8*, and *PPARγ* for exclusive caffeine exposure to THP-1 pre-monocyte cells did not differ relative to the control group. Data represented as mean + SEM with N = 7 and analyzed by one-way ANOVA repeated measures on ΔCt values.

Exclusive caffeine exposure to THP-1 pre-monocytes at 25, 50, 100, and 150 μM concentrations for 2.5 h without LPS did not significantly alter constitutive gene expression of TNF-α, NF-κB, IL-8, or PPARγ (Figure 1a–d).

### 3.2. Caffeine Administered After LPS Exposure (Treatment)

**Figure 2:**
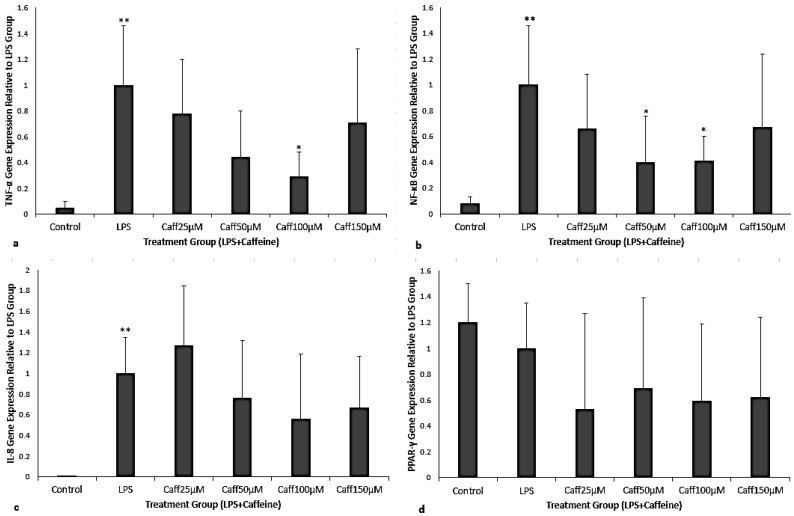
(**a**–**d**) Fold change of *TNF-α*, *NF-kB*, *IL-8*, and *PPARγ* gene expression relative to LPS exposure control ((**a**), (**b**), (**c**), and (**d**), respectively). The experimental groups consisted of control, LPS, and caffeine when given as treatment (25, 50, 100, 150 μM) after LPS. LPS exposure significantly increased *TNF-α*, *NF-kB*, and *IL-8* compared to unexposed control ((**a**–**c**); ** = *p* < 0.001). (**a**) Significant decrease in *TNF-α* gene expression at a caffeine dose of 100 μM (* *p* = 0.001) as treatment when compared to LPS exposure alone. (**b**) Significant decrease in *NF-κB* at 50 μM (* *p* = 0.04) and 100 μM (* *p* = 0.0024) caffeine concentrations when compared to LPS exposure alone. (**c**) *IL-8* gene expression with caffeine did not differ from LPS exposure alone. (**d**) *PPARγ* gene expression did not differ from LPS exposure alone. No significant change was noted with *PPARγ* gene expression regardless of the caffeine concentration. Data represented as mean + SEM with N = 7 and analyzed by one-way ANOVA repeated measures on ΔCt values.

THP-1 cells exposed to LPS significantly increased *TNF-α*, *NF-κB*, and *IL-8* mRNA but no change was detected in PPARγ gene expression compared to the control (Figure 2a–d, *p* < 0.001). After LPS exposure, there were dose-specific effects of caffeine in attenuating THP-1 inflammatory gene expression. When caffeine was administered 30 min post-LPS exposure there was a significant decrease in *TNF-α* gene expression, by 71% for 100 μM caffeine concentration (*p* = 0.001, Figure 2a). For *NF-κB* gene expression, there were decreases in gene expression by 60% and 59% for 50 and 100 μM caffeine concentrations, respectively (*p* = 0.04 and *p* = 0.024, respectively; Figure 2b). There were no significant changes for *IL-8* or *PPARγ* gene expression (Figure 2c,d).

### 3.3. Caffeine Administered Prior to LPS Exposure (Prophylaxis)

**Figure 3:**
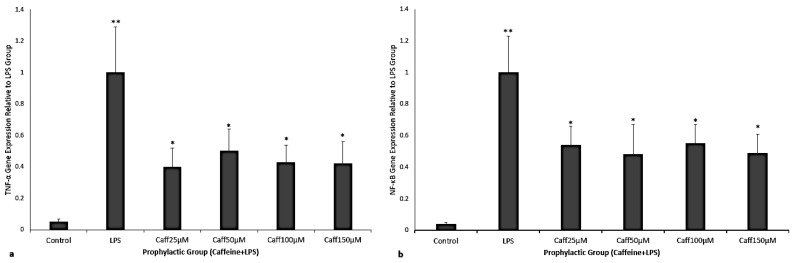
(**a**,**b**) Fold change in *TNF-α* and *NF-κB* gene expression relative to LPS-exposed group shown ((**a**), (**b**), respectively). The experimental groups consisted of control, LPS, and caffeine when given as prophylaxis (25, 50, 100, 150 μM) before LPS. LPS exposure significantly increased *TNF-α* and *NF-κB* compared to unexposed control ((**a**), (**b**), respectively; ** = *p* < 0.05). For all caffeine doses as prophylaxis to LPS-exposed cells when compared to LPS exposure alone there were significant decreases (* = *p* < 0.05) in *TNF-α* and *NF-κB* gene expression ((**a**), (**b**), respectively). Data represented as mean + SEM with N = 7 and analyzed by one-way ANOVA repeated measures on ΔCt values.

When caffeine was given as prophylaxis there was an attenuated expression of *TNF-α* and *NF-κB* for all concentrations of caffeine. There was a significant decrease in *TNF-α*, by 60%, 55%, 57%, and 58% for caffeine concentrations of 25, 50, 100, and 150 μM, respectively (*p* = 0.0034, *p* = 0.027, *p* = 0.046, *p* = 0.046, respectively; Figure 3a). There was a significant decrease in *NF-κB*, by 46%, 52%, 45%, and 51% for caffeine concentrations of 25, 50, 100, and 150 μM, respectively (*p* = 0.021, *p* = 0.011, *p* = 0.025, *p* = 0.031, respectively; Figure 3b).

### 3.4. Immunoassay

**Figure 4:**
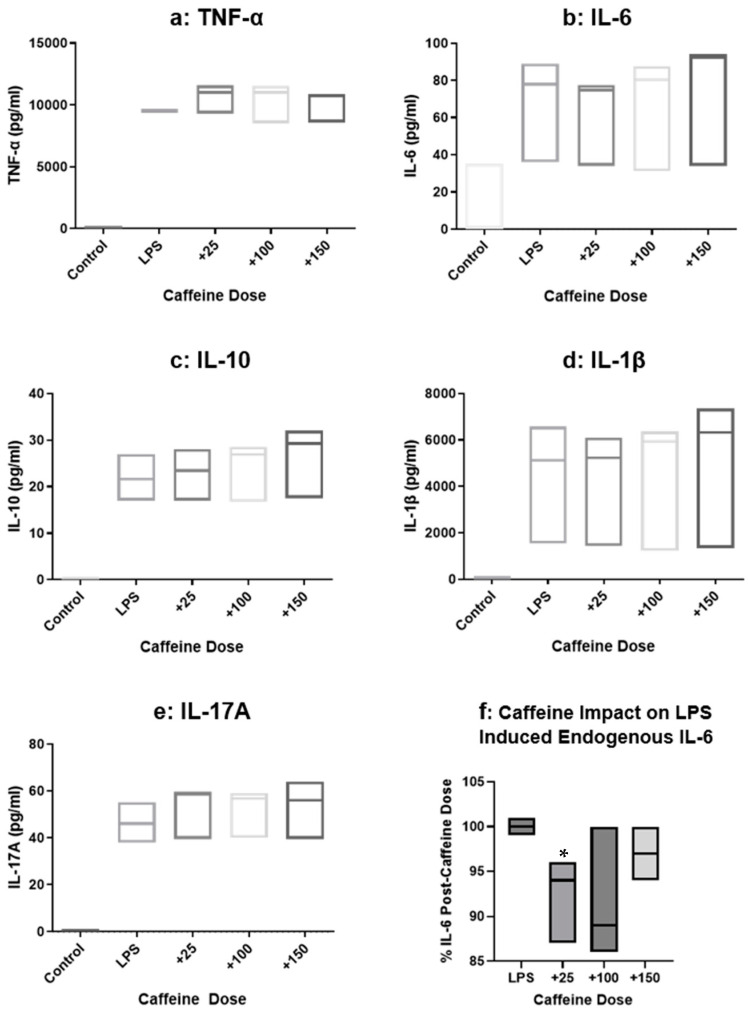
(**a**–**f**) Immunoassay for TNF-α, IL-6, IL-10, IL-1β, and IL-17. (**a**–**e**) No significant changes for cytokine productions with any of the caffeine doses. (**f**) Comparison of caffeine dose with LPS induced response showed a decrease in IL-6, * = *p* < 0.05.

For validation, TNF-α, IL-6, IL-10, IL-1β, and IL-17 analysis was done on the supernatants from our gene expression experiments. (Figure 4). Although slight changes in cytokines were observed with the titration of caffeine dosages, there was no statistical significance, implicating the impact of time on the translational response and protein secretion. The impact of caffeine on downstream protein is suggested by Figure 4f, with an implicated impact of caffeine on IL-6 response at 25 μM (*p* < 0.05, paired *t*-test, but not ANOVA). The variability and the short-time for translational/secretion prevented a clear understanding of the impact of caffeine on the synthesis, and secretion of the cytokines (including IL-6) in the monocytic cell line.

## 4. Discussion

Caffeine is a non-specific adenosine receptor antagonist which antagonizes the A1 and A2a adenosine receptors, neurologically stimulating ventilation at serum levels of 5–25 μg/mL in preterm infants [21,25]. Caffeine also possesses potent anti-inflammatory effects which may be of additive benefit during neonatal exposure to infection or other inflammatory stimulus through modulating pro-inflammatory gene expression [26,27,28]. These in vitro experiments assessed caffeine’s effect on macrophage LPS inflammatory response at the transcriptional level.

In our in vitro model, we demonstrated a dose-dependent capacity to decrease inflammatory gene expression in LPS-exposed THP-1 pre-monocytic cells. Although there have been previous studies assessing caffeine effects on THP-1 cells, our experimental approach focuses on the immediate pre-monocytic responsiveness to infection exposure using a gram-negative *Escherichia coli* bacterial analog LPS to interrogate inflammatory gene expression response in the presence and absence of caffeine. Transcription to translational secretion of protein was not performed at this point, since the time course of protein synthesis is significantly delayed post-transcriptionally to allow for synthesis and secretion. This is the focus of ongoing studies in our laboratory, which are the follow-up to these studies. We did not differentiate the THP-1 pre-monocytic cell line to reflect the immature immunity system of preterm infants to study caffeine prophylactic (pre-LPS) versus treatment (post-LPS) [29]. Polarization of monocytes/macrophages prior to LPS or caffeine exposure may lead to further upregulation of pro-inflammatory markers [30,31]. Because we were mimicking clinical scenario of a preterm infant, we wanted to assess how the caffeine as treatment and prophylactic would affect the monocytes response to a pro-inflammatory stimulus. Future studies can be done to assess if prophylactic caffeine promotes a specific monocyte differentiation or not. 

In the post-LPS group, we mimicked the clinical scenario of infants who were exposed in utero to chorioamnionitis and were given caffeine postnatally. The pre-LPS group or the prophylaxis group was intended to mimic a clinical scenario where preterm infants are universally given caffeine upon admission to the NICU and the infant has complications of late-onset sepsis with a gram-negative bacterium. Lastly, we used a dosing concentration range of 25 to 150 μM to reflect the clinical dosing of caffeine for primary indication of apnea in premature infants, that will lead to a serum level of 5 to 30 μg/mL.

Caffeine is administered to more than 90% of premature infants to prevent apnea of prematurity [32]. Premature infants are also at increased risk for infection and complications arising from increased inflammation [33]. Our study demonstrated that in addition to supporting respiratory drive, caffeine has a potential role in managing neonatal exposure to infection when administered as either simulated treatment or prophylaxis. While caffeine did not change the constitutive expression of the studied genes, the pre-monocytic cells pre-incubated with caffeine displayed attenuated LPS-induced *TNF-α* and *NF-κB* gene expression for all serum concentrations. This was also described in a previous study that utilized considerably higher caffeine concentrations, up to a maximum of 800 μM [14]. Our post-LPS caffeine group exhibited dose-dependent responses, with significant decreases in inflammatory gene expression with 50 and 100 μM caffeine concentrations, corresponding to serum levels of 10 and 20 μg/mL in preterm infants. This serum level in preterm infants is achieved when the current FDA-approved dosing of caffeine is administered [21,22]. These dose-dependent anti-inflammatory effects of caffeine may underlie a previous retrospective chart review study showing that caffeine concentrations >14.5 μg/mL were associated with reduced chronic lung disease in infants born at less than 29 weeks of gestation [18]. Interestingly, with a higher caffeine concentration of 150 μM, both *TNF-α* and *NF-κB* gene expression was no longer significantly decreased in our treatment model. In an observational prospective study by Chavez Valdez et al., the most significant decrease in pro-inflammatory cytokines of TNF-α, IL-1β, and IL-6 was with caffeine serum levels between 15 and 20 μg/mL [18].

In this in vitro study, caffeine, when given as treatment post-LPS exposure in non-differentiated pre-monocytic THP-1 cells, demonstrated an optimal dose for the attenuation of pro-inflammatory gene expression. A significant impact of caffeine was observed with the 50 and 100 μM caffeine concentrations. However, at the higher caffeine concentration of 150 μM, both *NF-κB* and *TNFα* gene expression no longer differed from pre-LPS alone. These observations are consistent with Chavez Valdez’s studies which showed that a caffeine concentration of 150 μM correlates to serum levels of 30 μg/mL and enhancement rather than attenuation of pro-inflammatory cytokine gene expression [16,18]. When caffeine was given prophylactically, before exposure to the gram-negative bacterial analog LPS, there was a significant decrease in both *NF-κB* and *TNFα* gene expression. Although serum levels may not be routinely checked in preterm infants to monitor for toxicity, there could be a potential benefit of measuring therapeutic serum levels of caffeine and pro-inflammatory cytokines in certain clinical scenarios. Lastly, the pre-LPS group (prophylaxis) showed that caffeine decreases the pro-inflammatory response of pre-monocytes to endotoxins. This may translate to a protective effect of caffeine in preterm infants by reducing inflammatory gene responses to an infectious insult.

Because of the dose-dependent effect observed with caffeine in our treatment model, additional genes were investigated. *IL-8* is a chemokine produced by macrophages in response to LPS to attract neutrophils. While LPS robustly induced *IL-8* gene expression in our macrophage preparations, we did not see a significant change in *IL-8* expression with caffeine treatment compared to LPS alone (Figure 2c). We also investigated whether the anti-inflammatory properties of caffeine may be regulated through induction of the anti-inflammatory master transcription factor PPARγ. We did not see any significant changes to *PPARγ* gene expression with LPS exposure or caffeine treatment (Figure 2d). We speculate that there was no significant change in either *IL-8* or *PPARγ* expression due to the short duration of caffeine exposure (2.5 h after LPS exposure for the treatment group). Another possibility could be that caffeine has no modulatory effect on IL-8 and may continue to elevate or prolong the LPS-induced IL-8 expression [34]. Lastly, we performed our experiment on undifferentiated monocytes; therefore, the expression of *PPARγ* may be more robust in matured macrophages [35].

Our in vitro model strengthens prior data focused on the anti-inflammatory benefits of caffeine. In a hyperoxia-based rat model, a caffeine dose of 10 mg/kg had anti-oxidative and anti-inflammatory effects on experimental oxygen-mediated lung injury [26,27]. In addition, there was a decrease in pro-inflammatory mediators and the redox-sensitive transcription factor NF-κB in the hyperoxia-exposed lung tissue of the caffeine-treated group compared to the non-treated group. These experiments suggest a protective effect of caffeine in the neonatal lung in both LPS and hyperoxia animal models that may be relevant in the development of BPD [9,26,27,28].

One of the limitations of this study is that THP-1 pre-monocyte cells may not have the same type of responses to LPS as compared to primary cells, human peripheral blood mononuclear cells (PBMCs), but the advantage of the differentiation state of THP-1 and homogeneity provides a good start to exploring the impact of caffeine on transcriptional regulation. Primary cell studies from neonates are a focus of ongoing collaborative studies, but the dosing may be different due to the lack of transformation and higher degree of differentiation. Another possible limitation is that we did not conduct cell viability studies at different caffeine exposures. However, a previous study that exposed differentiated THP-1 monocytes to a higher caffeine concentration range of 100–800 μM showed no significant impact on cell survival compared with a control [14]. Although we focused specifically on gene expression, the endpoint of protein secretion would imply the impact of caffeine on LPS-induced gene expression, and the efficiency of the secreted protein product. In our gene expression analysis, the protein tended towards changes but there was an obvious translational “time”, which would be required to be established downstream. This is the focus of ongoing work in our laboratory. The follow-up on mechanistic interrogation of the unique prophylactic and post-LPS differences we observed with different caffeine doses will be important to determine the regulatory relationship between the gene expression and protein with caffeine exposure. These studies were all performed with the gram-negative bacterial analog, LPS. LPS generally mimics a gram-negative bacterial exposure, which compromises 94% of chorioamnionitis in preterm infants and 36.6% of culture-positive early-onset sepsis in preterm infants [36,37]. Therefore, our study may not reflect late-onset sepsis in preterm infants caused by gram-positive bacteria such as coagulase-negative staphylococcus [38,39].

Future studies could determine whether the differences between pre- and post-LPS groups would have the same effect on *Staphylococcus aureus* gram-positive bacteria analogs such as peptidoglycan. Late-onset sepsis in preterm infants involves gram-positive bacteria such as coagulase-negative staphylococcus and is just as prevalent as gram-negative infections [35,36]. Importantly, future studies would also include whole-bacteria exposures both in vitro and in vivo, since the gram-negative and gram-positive analogs represent only one aspect of the bacteria that induces inflammation. In addition, a time-course experiment could be studied to observe the effects of caffeine at different time points, particularly over longer periods, assessing whether the anti-inflammatory effects are sustained or transient. Gene expression precedes translation and secretion of protein defining the paracrine repertoire of caffeine therapeutic efficacy. Defining the relationship between the transcriptional gene expression response demonstrated in this manuscript followed by a time course protein study can also assess the relationship between the transcription and translation of secreted products. Lastly, the prophylactic protective effect of caffeine could also be studied in the NLRP3 inflammasome pathway, which is regulated by adenosine receptors [14,40].

## 5. Conclusions

The findings from this in vitro LPS-stimulated human pre-monocyte model showed that caffeine can mitigate pro-inflammatory responses in macrophages. The prophylactic use of caffeine showed a protective effect for pre-monocytes at all the studied caffeine concentrations. Caffeine had a dose-dependent response in attenuating inflammatory genes when administered as treatment following LPS. This has important implications for caffeine dosing in preterm infants that can be translated into improved algorithms for caffeine administration, aimed at optimizing anti-inflammatory efficacy. Further, these findings may indicate a benefit of caffeine for regulating monocyte inflammatory responses to gram-negative infections.

## Data Availability

All data generated or analyzed during this study are included in this article. Further inquiries can be directed to the corresponding author, Zeyar Htun (zeyar.htun@nyulangone.org).

## Abbreviations

The following abbreviations are used in this manuscript:

NICU	Neonatal intensive care unit
BPD	Bronchopulmonary dysplasia
LPS	Lipopolysaccharide
RT-PCR	Reverse transcriptase-polymerase chain reaction
PMA	Phorbol myristate acetate
